# Lower trapezius transfer with semitendinosus tendon augmentation: Indication, technique, results

**DOI:** 10.1007/s11678-018-0495-8

**Published:** 2018-11-20

**Authors:** Philippe Valenti, Jean-David Werthel

**Affiliations:** 1 Shoulder Unit, Clinique Bizet, 75116 Paris, France; 2 Department of Orthopedic Surgery, Hôpital Ambroise Paré, 92100 Boulogne-Billancourt, France

**Keywords:** Arthroscopy, Shoulder, Rotator cuff tears, Lower trapezius transfer, Lack of active external rotation, Arthroskopie, Schulter, Rotatorenmanschettenrisse, Transfer des unteren Anteils des M. trapezius, Verminderte aktive Außenrotation

## Abstract

**Background:**

Lower trapezius transfer can restore external rotation in brachial plexus palsies. In some cuff tear arthropathies, there is lack of active external rotation with a preservation of forward elevation. We evaluated the clinical outcomes of a lower trapezius transfer extended with a semitendinosus tendon and fixed to the insertion of the infraspinatus via arthroscopy.

**Methods:**

Between 2013 and 2016, we operated on 14 patients (8 men, 6 women; mean age of 62 years, range: 50–70) to reconstruct irreparable posterosuperior rotator cuff tear. A vertical incision of 6 cm following the medial border of the spine was made to harvest the lower trapezius in extension with the semitendinosus tendon. The extension band of the lower trapezius was fixed laterally via arthroscopy on the great tuberosity at the level of the insertion of the infraspinatus. The proximal stump of this extension band was then fixed medially into the muscle of the lower trapezius with the arm in maximum external rotation. Outcomes were evaluated with the Constant–Murley score, simple shoulder test (SST), and subjective shoulder value (SSV).

**Results:**

Over a mean follow-up of 24 months (range: 12–36 months), the gain in external rotation with the arm at the side was 24° and 40° in 90° of abduction. The Constant–Murley score improved from 35 to 60 points, the SST from 3.5 to 7.5, the SSV from 30 to 60%, and the pain decreased from 7 to 2 (visual analogue scale, 0–10). Both the lag sign and hornblower sign were negative after this transfer. There were two cases of hematomas, and one was revised because of infection.

**Conclusion:**

Lower trapezius transfer is a therapeutic option for irreparable posterosuperior cuff tears with a lack of active external rotation and a good subscapularis. Patients can expect improvements in pain and in active external rotation without any loss of active anterior elevation.

## Zusammenfassung

**Hintergrund:**

Bei brachialen Plexuslähmungen kann ein Transfer des unteren Anteils des M. trapezius die Außenrotation wiederherstellen. Bei einigen manschettenrupturbedingten Arthropathien fehlt es an aktiver Außenrotation, während die Vorwärtshebung erhalten ist. Evaluiert wurden die klinischen Ergebnisse nach Transfer des mit einer Semitendinosus-Sehne verlängerten und arthroskopisch an den Ansatz des M. infraspinatus fixierten unteren Trapeziusanteils.

**Methoden:**

Zwischen 2013 und 2016 wurden 14 Patienten (8 Männer, 6 Frauen; Durchschnittsalter 62 [50–70] Jahre) mit irreparabler posterosuperiorer Rotatorenmanschettenruptur rekonstruktiv operiert. Über einen 6 cm langen Schnitt am medialen Wirbelsäulenrand wurde der untere Anteil des M. trapezius in Verlängerung mit der Semitendinosussehne mobilisiert. Das „Verlängerungsband“ des unteren M. trapezius wurde arthroskopisch seitlich an der Tuberositas maior in Höhe des Ansatzes des M. infraspinatus fixiert. Der proximale Stumpf dieses Bandes wurde dann medial in den unteren Trapeziusanteil fixiert mit dem Arm in maximaler Außenrotation. Anhand des Constant-Murley-Scores, des SST („simple shoulder test“) und des SSV („subjective shoulder value“) wurde das Outcome evaluiert.

**Ergebnisse:**

Nach einer durchschnittlichen Follow-up-Zeit von 24 (12–36) Monaten betrug der Funktionszuwachs hinsichtlich der Außenrotation bei an der Seite gehaltenem Arm in 90°-Abduktion 24° und 40°. Der Constant-Murley-Score hatte sich von 35 auf 60 Punkte verändert, der SST von 3,5 auf 7,5, der SSV von 30 auf 60 % und die Schmerzen von 7 auf 2 (visuelle Analogskala, 0–10). Nach dem Transfer waren sowohl das Lag- als auch das Hornblower-Zeichen negativ. Bei 2 Patienten kam es zu Hämatomen, bei einem zu einer infektionsbedingen Revision.

**Fazit:**

Ein unterer Trapezius-Transfer ist eine therapeutische Option bei posterosuperiorer Rotatorenmanschettenruptur mit eingeschränkter aktiver Außenrotation und einer guten Subscapularis-Situation. Zu erwarten sind eine Schmerzlinderung und eine verbesserte aktive Außenrotation bei unveränderter aktiver Vorwärtshebung

Lower trapezius transfer has been used to restore external rotation in lesions of the brachial plexus, with promising results published by, among others, Elhassan et al. [1] and Bertelli et al. [2]. In 2009, Elhassan et al. [1] reported the results of a lower trapezius transfer in a 55-year-old man with traumatic brachial plexus palsy. This transfer improved the patient’s active external rotation from inability to 30° at the 9‑month follow-up. Since then, this technique has been performed in many other cases [3] offering satisfactory results concerning external rotation. Duncan et al. in 2014 [4] proposed to widen the indication to include massive irreparable posterosuperior cuff tears with a lack of active external rotation.

Lower trapezius transfer restores external rotation in brachial plexus injury

In 2016, Elhassan et al. [5] reported the outcomes of 33 patients treated with lower trapezius transfer for reconstruction of symptomatic irreparable rotator cuff tears. At the final follow-up of nearly 4 years, there was significant improvement; patients who had a preoperative flexion of >60° achieved more significant gains in their range of motion. To reach the insertion of the infraspinatus on the greater tuberosity, the authors proposed to use an Achilles tendon allograft to extend the length of the lower trapezius tendon.

To avoid the potential complications associated with an allograft, we propose harvesting the semitendinosus tendon from the lower limb to augment the length of the lower trapezius and to reach the infraspinatus insertion. Fixation is performed arthroscopically on the greater tuberosity of the humeral head and the autograft is tensioned medially at the level of the tendinous portion of the lower trapezius using a Pulvertaft suture technique.

The present study reports the outcome of lower trapezius transfer prolonged with a semitendinosus autograft to reconstruct massive irreparable posterosuperior rotator cuff tear.

## Patients and methods

We report the outcome of a monocentric prospective study comprising 15 patients who underwent lower trapezius transfer between 2013 and 2016 to reconstruct irreparable posterosuperior rotator cuff tear with at least 1 year of follow-up.

### Inclusion and exclusion criteria

The indications for surgery included all four of the following criteria:

1. A lack of active external rotation with the arm at the side, a hornblower sign, limitation in active abduction and forward elevation, and persistent pain and weakness of the shoulder
2. Magnetic resonance imaging (MRI) demonstrating a massive irreparable tear of the posterosuperior rotator cuff with atrophy of the teres minor and of the infraspinatus and fatty infiltration (> grade 2 Goutallier classification)
3. Failed conservative treatment
4. Full passive range of motion and patient motivated for postoperative immobilization (6 weeks) and intensive physiotherapy (6 months)

Patients were not eligible for this procedure if they had:

1. An active forward elevation of ≤80° with an anterosuperior escape of the humeral head (pseudoparalytic shoulder)
2. An associated subscapularis tear (> grade II Lafosse classification)
3. Glenohumeral arthritis
4. A deltoid palsy

### Surgical technique

**Fig. 1:**
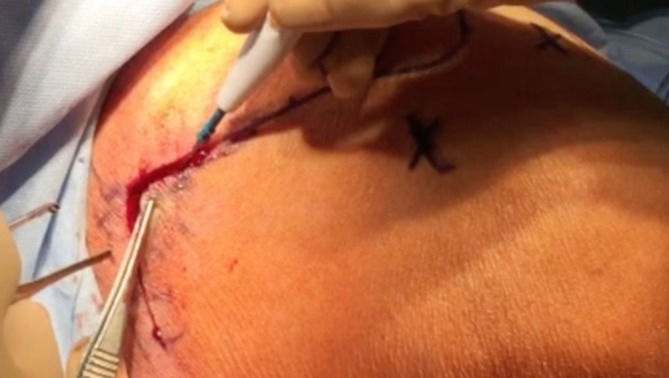
Mini-invasive vertical posterior approach (6 cm) with posterior and lateral portals for arthroscopic exploration of the shoulder and partial repair and fixation of the semitendinosus tendon

**Fig. 2:**
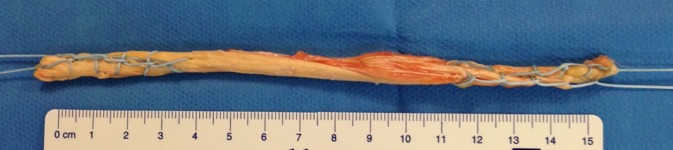
Semitendinosus tendon doubled and reinforced with a distal and proximal Krackow suture and a nonabsorbable Orthocord #2 (DePuy Mitek, Raynham, MA, USA); length between 10 and 15 cm

**Fig. 3:**
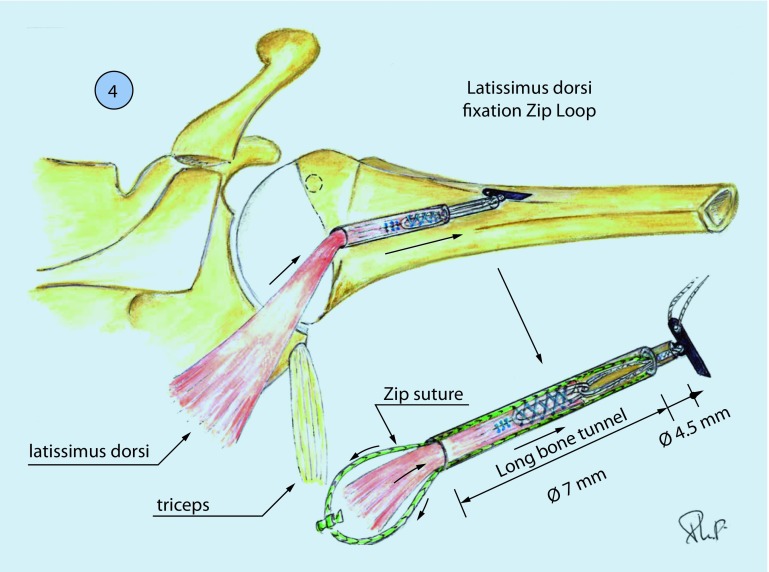
The semitendinosus is introduced at the level of the insertion of the infraspinatus into an anteroposterior bone tunnel and locked with a ZipTight device

**Fig. 4:**
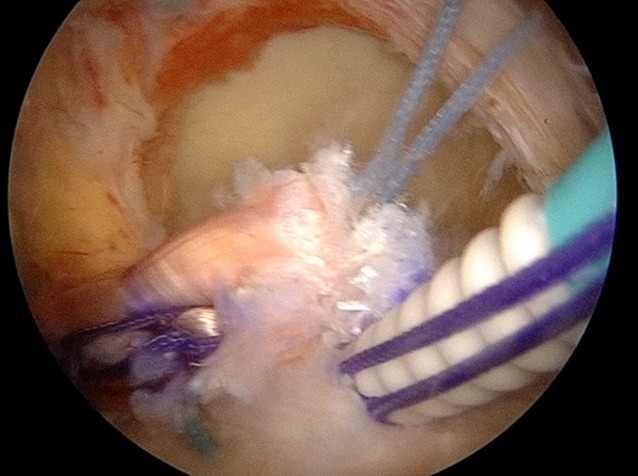
The semitendinosus tendon is first fixed by arthroscopy at the level of the insertion of the infraspinatus on the footprint (after shaving this area) with two or three anchors for good adaptation of the tendon to the bone

**Fig. 5:**
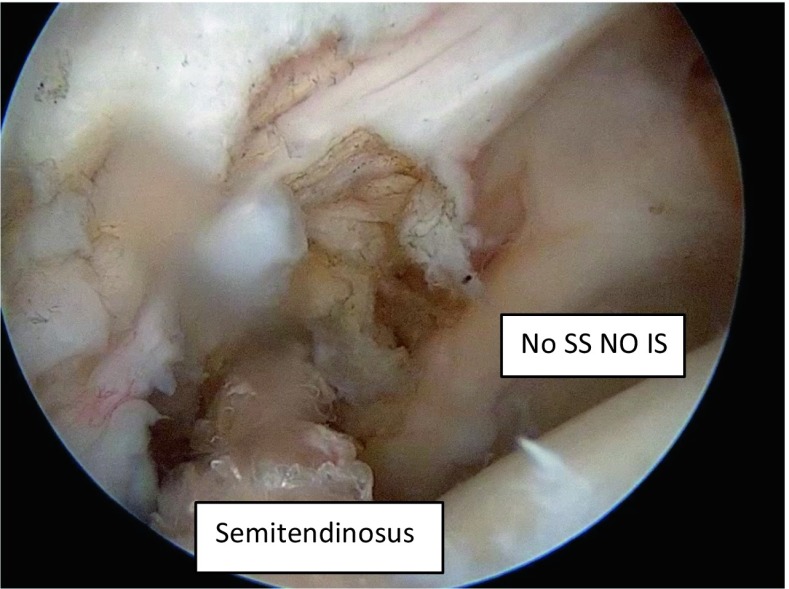
In a massive posterosuperior cuff tear the semitendinosus graft is fixed to the footprint to reproduce the direction of the infraspinatus. *SS* supra spinatus, *IS* infraspinatus

**Fig. 6:**
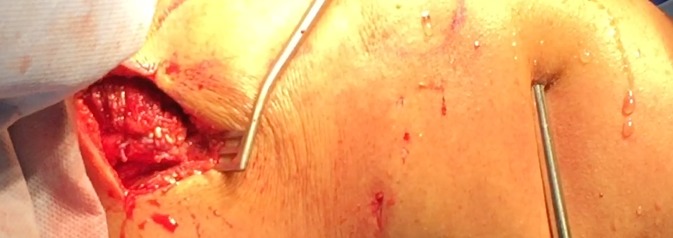
With the shoulder in 60° external rotation and 30° abduction, the medial stump of the graft is passed through the short tendon and sutured to itself with a nonabsorbable suture (Orthocord #2; DePuy Mitek, Raynham, MA, USA). A Krackow suture is placed in the trapezius and into the semitendinosus tendon to reinforce the repair

The surgical technique is illustrated in Figs. 1, 2, 3, 4, 5 and 6.

All surgeries are performed with the patient under general anesthesia with an interscalene block. The patient is positioned in the beach chair position and the first step involves diagnostic arthroscopy to confirm the irreparability of the posterosuperior cuff. Debridement of the subacromial space is performed with a radiofrequency ablation device. Tenotomy or tenodesis of the long head of the biceps is carried out. The coraco-acromial ligament is preserved and a smooth acromioplasty is performed if an acromial spur is found intraoperatively.

#### Arthroscopic preparation

In all cases, the supraspinatus and the infraspinatus were retracted at the level of the glenoid and their reinsertion to the humeral head (even medially) was not possible even after an extensive release. The subscapularis is confirmed to be intact. In cases where a tear of the superior third of the subscapularis (grade I or II in the Lafosse classification) was found, a suture bridge repair was performed. The greater tuberosity at the level of the infraspinatus footprint is then prepared and the bone is smoothly freshened with a burr.

#### Lower trapezius harvest

A mini-invasive vertical posterior approach (Fig. 1) is made starting 2 cm medially to the deltoid tubercle of the spine of the scapula and extending distally to about 5 cm. The inferior border of the lower trapezius is released and separated from the underlying fascia of the infraspinatus and the lower trapezius triangular tendinous insertion is identified. The trapezius is then split progressively horizontally and medially to divide the lower and the middle trapezius. The spinal accessory nerve runs vertically on the deep surface of the trapezius, 2 cm medially to the spinal border of the scapula. Care should be taken not to injure the nerve during the split, which should not be extended too medially. A nerve stimulator can be used to confirm the location and the normal function of the spinal accessory nerve. The tendon is detached from its insertion. It is a short tendon and care is taken to harvest the maximal tendon length possible. The tendon is then reinforced via a Krackow technique with a non-absorbable suture placed into the tendinous and musculotendinous portions of the lower trapezius to facilitate its mobilization.

#### Semitendinosus harvest

A 20-cm semitendinosus autograft is then harvested with a stripper and two small incisions. This tendon is folded in two to obtain a length of about 10 cm. A Krackow suture is performed with a braided non-absorbable suture for reinforcement and tubulization of the semitendinosus tendon.

#### Fixation of the transfer

A deep subcutaneous tunnel is created from the medial dorsal incision to reach inside the glenohumeral joint. Blunt scissors are used to create a passage between the posterior deltoid and the atrophic infraspinatus.

The patient can return to unrestricted activities after 6 months

Two different techniques of fixation on the humeral head were used in this series:

1. The semitendinosus graft was first sutured medially to the tendon of the lower trapezius in a Pulvertaft fashion. A cortical button (ZipTight, Biomet Zimmer, Warsaw, IN, USA) was then sutured to the lateral free end of the graft. Under arthroscopic visualization, a bone tunnel was created from posterior of the infraspinatus footprint to the bicipital groove anteriorly using a guiding device. The cortical button was then shuttled from the medial skin incision to the glenohumeral joint through the passage previously created under lateral arthroscopic visualization; it was then shuttled into the bone tunnel. The patient’s arm was positioned at 60° of external rotation and 30° of abduction and the strands of the cortical button were tightened to introduce 2–3 cm of the graft into the tunnel.
2. The semitendinosus tendon was fixed first by arthroscopy at the level of the insertion of the infraspinatus on the footprint (after shaving this area) with two or three anchors for good adaptation of the tendon to the bone. The medial stump of the semitendinosus graft was pushed to the subposterior deltoid space following the direction of the infraspinatus to reach the medial tubercle of the scapula. This tunnel should be large to achieve perfect gliding of the semitendinosus tendon and to avoid adhesions. Thus, with the shoulder in 60° external rotation and 30° abduction, the medial stump of the graft is passed through the short tendon and sutured to itself with a nonabsorbable suture (Orthocord #2; DePuy Mitek, Raynham, MA, USA). A Krackow suture is placed in the trapezius and into the semitendinosus tendon to reinforce the repair.

### Postoperative care

Postoperatively, patients were placed in a custom-made shoulder brace in 30° of abduction and 30° of external rotation all day and night for 6 weeks day and then only at night for the following 4 weeks. After 6 weeks, the patients started pain-free passive and active assisted range of motion exercises in elevation and external rotation. After 3 months, full active range of motion exercises were started along with gentle strengthening. After 6 months the patient is allowed to return to unrestricted activities.

### Clinical and radiological evaluation

**Fig. 7:**
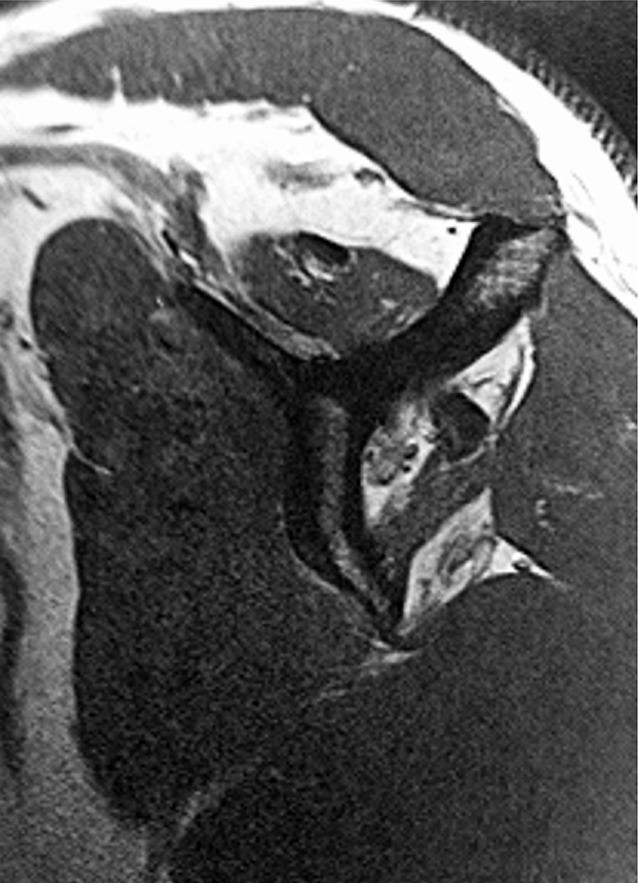
Magnetic resonance image showing atrophy of the supraspinatus, infraspinatus, and teres minor and an excellent subscapularis; clinically the patient has a lag sign and a drop sign and forward elevation is complete

**Fig. 8:**
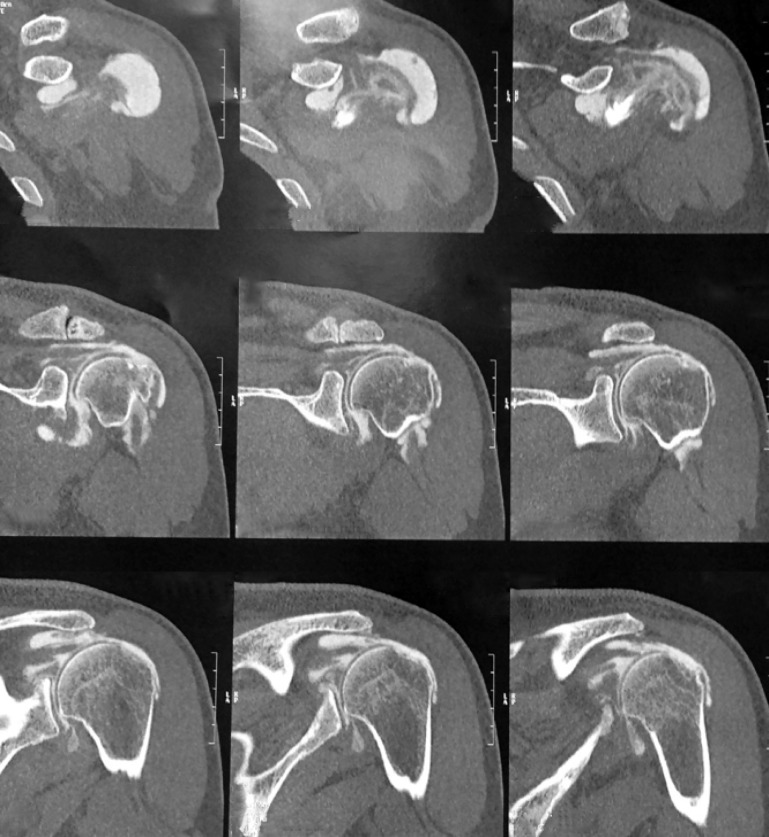
No osteoarthritis with narrowing of the subacromial space

Clinical outcome measures included pain levels, shoulder range of motion, shoulder subjective value (SSV), and Constant–Murley scores. Pain was quantified with the visual analog scale (VAS). Range of motion was measured using a standard goniometer. Internal rotation was assessed by the most cephalic vertebral segment reached by the thumb (Figs. 7 and 8).

Patients had a preoperative standard radiological evaluation of the shoulder to analyze the height of the subacromial space and the grade of osteoarthritis according to Hamada et al. [6]. All patients had an acromiohumeral distance of <5 mm before surgery, but with no signs of glenohumeral osteoarthritis and no evidence of acetabulization (Hamada <3). Magnetic resonance imaging was performed preoperatively for all patients to assess atrophy according to Thomazeau et al. [7] and/or fatty infiltration of the subscapularis, supraspinatus, infraspinatus, and teres minor according to Goutallier et al. [8].

## Results

**Fig. 9:**
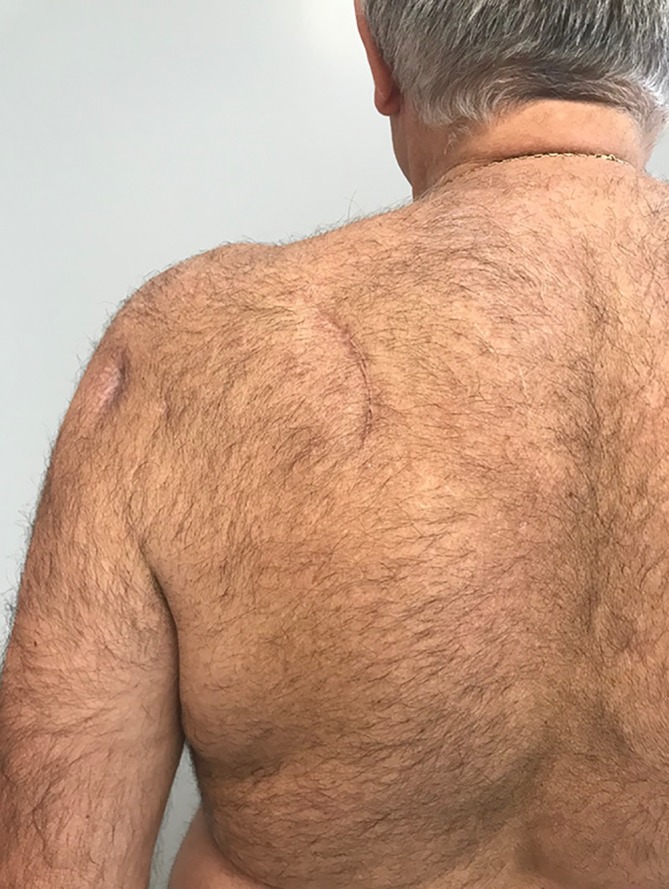
Vertical mini-invasive posterior approach over the tubercle of the scapula to identify the lower trapezius with posterior and lateral portals for fixation by arthroscopy on the footprint

**Fig. 10:**
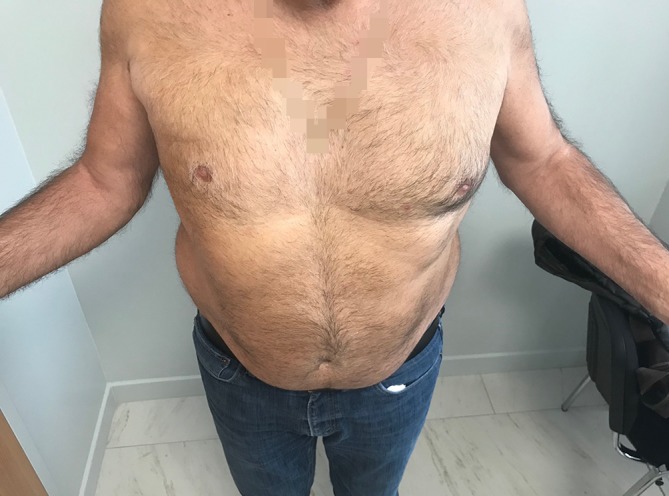
Active external rotation on the left side, with the arm at the side at 30°; no lag sign

**Fig. 11:**
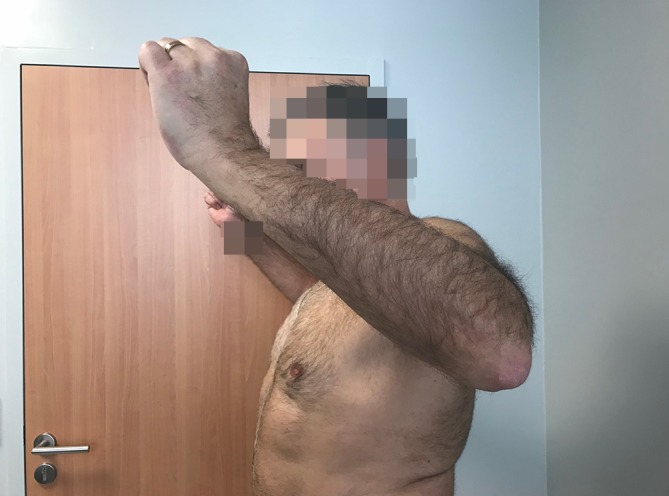
Active external rotation in abduction (30°); no hornblower sign

**Fig. 12:**
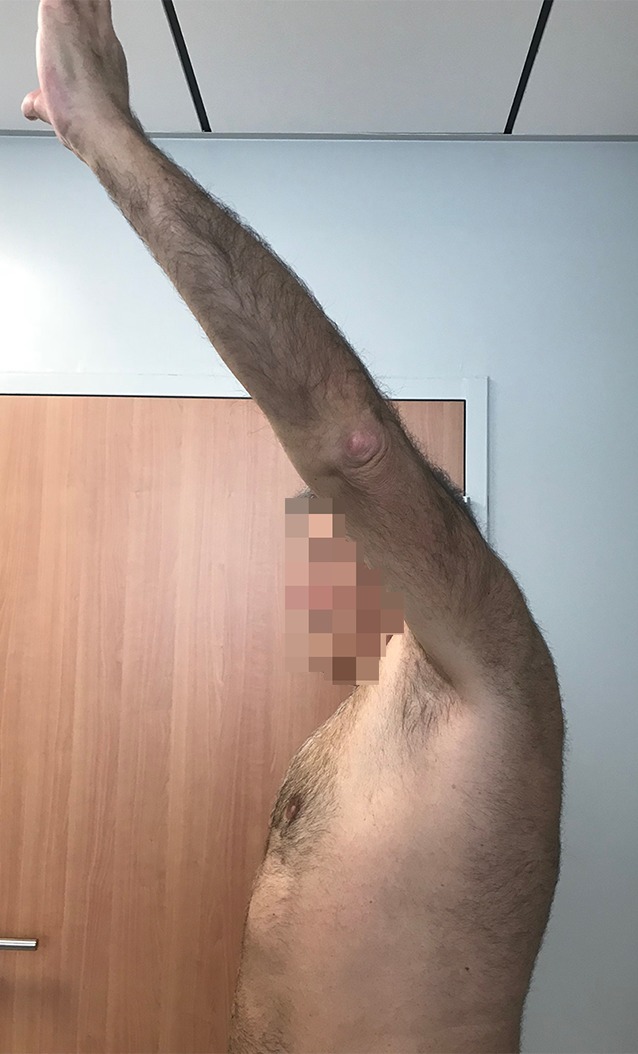
Complete forward elevation (170°)

**Fig. 13:**
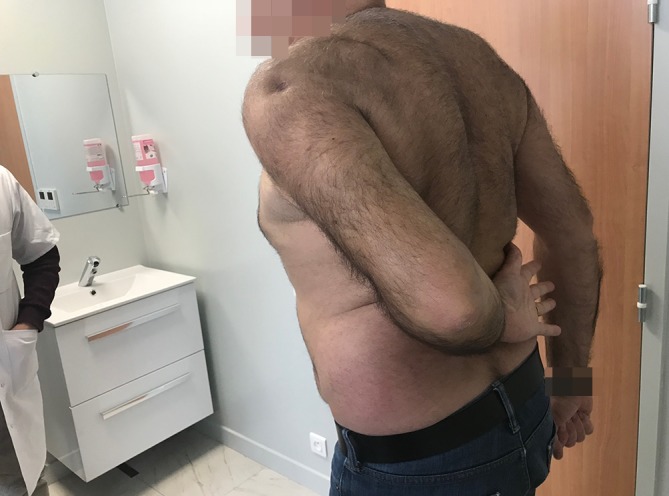
No limitation in medial rotation with the thumb at the level of L1

The results of the tendon transfer technique used in this study are shown in Figs. 9, 10, 11, 12 and 13.

### Study population characteristics

The study population comprised eight men and six women, with an average age of 62 years (range, 50–70 years). Symptoms included progressively worsening shoulder pain, weakness particularly in external rotation with the arm at the side and in abduction, and limitations in shoulder elevation. Nine patients had undergone prior surgery (arthroscopic rotator cuff repair [6]) and were referred to our clinic to be evaluated for potential tendon transfer. Five patients had no prior surgery (three posterosuperior irreparable cuff tear and two cases of a myotendinous lesion of the infraspinatus [9]). We performed an isolated arthroscopically assisted lower trapezius tendon transfer in ten cases. In four patients who had a preoperative active elevation of <120°, the transfer was combined to a latissimus dorsi transfer.

### Preoperative clinical evaluation

Preoperatively, all patients showed variable degrees of loss of active shoulder function, with average shoulder flexion of 150° (range, 100°–180°), external rotation with the arm at the side of −20° (range, −50°–0°), external rotation in 90° of abduction of −10° (range, −30°–20°), and internal rotation to L3 (range, L5–T10).

All patients had a positive external rotation lag sign with the arm elevated at 20° in the plane of the scapula and a positive external rotation lag sign with the arm at 90°of abduction [10]. Results of the belly-press test and lift-off test were normal in all patients. The main complaint was a weakness in external rotation. Preoperatively, the mean VAS was 7 (range, 2–10), the mean SSV was 40% (range, 10–60), and the mean absolute Constant–Murley score was 35 (range, 20–50).

### Postoperative clinical evaluation

All patients were reviewed at a mean follow-up of 24 months (range, 18–36 months). Mean active forward flexion improved from 150° to 160°, external rotation with the arm at the side improved from −20° to 24°, and external rotation with the arm at 90° of abduction improved from −10° to 40°. The mean Constant–Murley score improved from 35 ± 15 to 60 ± 9. Mean VAS decreased from 7 to 2, and mean SSV improved from 40 to 70%. All these changes were statistically significant (*p* <0.001). Postoperatively, the lag sign and the drop sign were negative in all patients.

We did not observe any difference when the lower trapezius transfer was isolated or combined with the latissimus dorsi.

### Complications

Two complications were noted in this series. Two patients had revision surgery for hematoma localized on the harvest site. Of these two patients, one had *Cutibacterium acnes* infection and was treated by open debridement and oral antibiotics. These two patients healed uneventfully and obtained a satisfactory outcome.

## Discussion

This study shows that arthroscopically assisted lower trapezius transfer elongated with an autograft can restore active external rotation with the arm at the side and the arm in abduction. This procedure offers satisfactory functional results in the treatment of irreparable posterosuperior cuff tears with atrophy and fatty infiltration of the infraspinatus and teres minor.

Our results are in agreement with those of Elhassan et al. published in 2016 [5]. In their series of 33 patients, the authors used an Achilles tendon allograft to elongate the lower trapezius. The fixation of this allograft was made through an open incision with an osteotomy of the acromion and with anchors under tension to the footprint of the supraspinatus and to the upper footprint of the infraspinatus. One third of their patients had a functional teres minor and all the patients had an irreparable supra- and infraspinatus tear. One third of the patients had an associated tear of the upper part of the subscapularis. The tear of the upper part of the subscapularis was repaired when present and the infraspinatus was advanced medially after release to reduce the size of the rupture. At an average follow-up of 47 months, 32 of the 33 patients had significant improvement in pain levels and in range of motion of the shoulder, especially in external rotation and in forward flexion. The authors concluded that the lower trapezius tendon transfer elongated with an Achilles allograft may lead to good outcome in most patients, especially for those who have a preoperative flexion of >60°.

The most popular tendon transfer traditionally used for irreparable posterosuperior tears with or without teres minor has been the latissimus dorsi transfer. The reported results are satisfactory in terms of pain relief, but not always satisfactory in terms of shoulder function particularly in restoring active external rotation with the arm at the side when the teres minor is atrophic or torn [11–15]. This could be explained biomechanically. In 1999, Herzberg et al. [16] demonstrated that the latissimus dorsi has a greater excursion but less strength than the infraspinatus and teres minor muscles. In addition, the latissimus dorsi transfer is always posterior and inferior to the infraspinatus and teres minor muscle bellies, resulting in abnormal vectors across the glenohumeral joint and therefore abnormal kinematics [17]. Recent biomechanics papers have proven the superiority of the lower trapezius transfer; Omid et al. in 2015 [18] showed that the orientation of the lower trapezius provided better anteroposterior balancing force and restored compressive forces closer to normal with improved glenohumeral kinematics compared with the latissimus dorsi. In 2012, Hartzler et al. [19] showed that the lower trapezius transfer is more effective in restoring external rotation with the arm at the side than with the arm at 90° of abduction. In addition, the lower trapezius acts as an agonist in external rotation. An electromyographic study showed that the lower trapezius is activated during external rotation [20].

### Limitations

This study has some limitations. Firstly, it comprised a small series with no control group and with a short follow-up. However, it is the first study to report the results of arthroscopically assisted lower trapezius transfer elongated with an autograft to restore active external rotation when both the teres minor and infraspinatus are deficient.

## Practical conclusion

- This study confirms that arthroscopically assisted lower trapezius transfer elongated with an autograft leads to improved Constant score, range of motion, strength, pain scores (VAS), and SSV score for irreparable supraspinatus, infraspinatus, and teres minor tears.
- This is an effective technique for relieving pain and restoring active external rotation.
- Longer follow-up is required to confirm the durability of this tendon transfer.
